# Dietary fat type modulates fatty acid digestibility, *de novo* lipogenesis, and energy expenditure in broiler chickens

**DOI:** 10.1016/j.psj.2026.107073

**Published:** 2026-05-05

**Authors:** Sasiphan Wongsuthavas, Metha Wanapat, Chalermpon Yuangklang, Kraisit Vasupen, Wannaree Wongtrairat, Benjamad Khonkhaeng, Jiravan Khotsakdee, Anton C. Beynen

**Affiliations:** a Department of Animal Science, Faculty of Agricultural Innovation and Technology, Rajamangala University of Technology Isan, Nakhon Ratchasima 30000, Thailand; b Faculty of Animal Science, Universitas Brawijaya, Indonesia; c Department of Animal Science, Faculty of Agriculture, Khon Kaen University, Khon Kaen 40002, Thailand; d Department of Electronics Engineering, Faculty of Engineering and Technology, Rajamangala University of Technology Isan, Thailand; e Department of Clinical Sciences of Companion Animals, Faculty of Veterinary Medicine, Utrecht University, Utrecht, the Netherlands (Retired)

**Keywords:** Broiler chickens, Fatty acid digestibility, Energy expenditure, Dietary fat, Krabok oil

## Abstract

This study tested the hypothesis that krabok oil (KO), a medium-chain saturated fatty acid (MCT)-rich fat source, would mimic the growth and carcass fat reducing effects of polyunsaturated fatty acid (PUFA)-rich soybean oil (SBO) in broiler chickens. Forty-five 7-day-old Arbor Acres broilers were randomly assigned to three dietary treatments tallow (T), SBO, and KO with 15 birds per treatment (*n* = 15) serving as individual replicates. Each iso-energetic diet was supplemented with 3.00 % of the respective fat source for 21-days (day 7 to day 28 of age). The results showed that KO-fed broilers achieved significantly greater final body weight (839.47 g vs. 750.60 g; *P* < 0.05) and average daily gain (32.84 g/d vs. 28.60 g/d; *P* < 0.05) compared to SBO-fed birds. However, the hypothesis was only partially supported; KO did not reduce abdominal fat (1.58 % vs. 1.04 % for SBO; *P* < 0.05), which was associated with the highest body energy retention in the KO group (37.33 % of intake). SBO significantly increased energy expenditure (44.97 % vs. 33.31 % of intake; *P* < 0.01) via enhanced PUFA-induced thermogenesis, whereas KO improved the digestibility of medium-chain fatty acids (e.g., lauric acid: 94.94 %; *P* < 0.01) without lowering total fat retention, likely due to its high saturated fatty acid (SFA) content favoring energy storage. Serum triglyceride concentrations were significantly lower in both SBO- and KO-fed birds (*P* < 0.01) compared to the tallow group, indicating active lipid catabolism. In conclusion, while KO serves as a potent growth promoter through efficient MCT utilization, it does not metabolically mimic the fat-reducing effects of SBO due to the interaction between chain length and saturation, which priorities energy partitioning toward retention rather than expenditure. These findings offer KO as a stable lipid alternative for optimizing broiler performance while clarifying the distinct metabolic roles of different fatty acid profiles.

## Introduction

Dietary fat is a concentrated source of energy in broiler nutrition and exerts significant effects on growth performance, nutrient utilization, and carcass composition. The physiological responses of poultry to dietary fat depend largely on its fatty acid profile, particularly the degree of saturation and chain length. Long-chain saturated fatty acids (SFA), such as those abundant in beef tallow, are less efficiently oxidized and tend to promote fat deposition in broiler carcasses (Sanz et al., 1999; Crespo and Esteve-Garcia, 2002). In contrast, polyunsaturated fatty acids (PUFA), which predominate in vegetable oils like soybean oil, have been shown to enhance fat digestibility and reduce fat accumulation by promoting fatty acid oxidation and altering lipid metabolism (Crespo and Esteve-Garcia, 2002; Villaverde et al., 2005; Zhou et al., 2021).

Medium-chain triacylglycerols (MCT), containing fatty acids with 6–12 carbon atoms, possess distinct metabolic properties compared with long-chain fatty acids. MCTs are rapidly hydrolyzed, absorbed directly into the portal circulation, and transported to the liver, where they are preferentially oxidized for energy rather than stored in adipose tissue (Bach and Babayan, 1982; Chen et al., 2018). This metabolic pathway increases energy expenditure and reduces fat deposition. In poultry, MCT supplementation has been associated with decreased abdominal fat and improved feed efficiency by shifting energy metabolism toward oxidation rather than storage (Zhao et al., 2022).

Krabok (*Irvingia malayana* Oliv. ex A. Benn.) is a tropical tree widely distributed in Southeast Asia whose seeds yield an oil traditionally consumed by humans and used in local cooking. Krabok oil (KO) is naturally rich in medium-chain saturated fatty acids, particularly lauric acid (C12:0) and myristic acid (C14:0) (Peangpra, 1977). While KO has been documented for culinary and traditional uses in humans, its nutritional potential as an MCT-rich dietary fat source in poultry production has received little scientific attention.

Furthermore, KO’s oxidative stability is critical for the feed industry. Due to its high saturated fatty acids and natural tocochromanols (Sonwai and Ponprachanuvut, 2012), KO exhibits remarkable resistance to rancidity. Since it is extracted from the seed kernel with its thin brown seed coat (testa), the oil benefits from seed coat-derived phytochemicals and total phenolic compounds. These provide potent antioxidant activities that combat oxidative stress and tissue damage (Sutthidech et al., 2024). This inherent stability ensures long-term storage and transport without degradation, making KO a viable, sustainable alternative for large-scale animal feed production.

The present study was conducted to compare the effects of three dietary fat sources tallow (high in long-chain SFA), soybean oil (rich in PUFA), and krabok oil (rich in medium-chain MCT) on growth performance, carcass energy retention, fat digestibility, and carcass fat deposition in broiler chickens. It was hypothesized that dietary KO would reduce carcass fat accumulation and enhance energy utilization through increased fatty acid oxidation, producing effects comparable to those of PUFA-rich soybean oil.

## Materials and methods

### Ethics approval

All experimental procedures were reviewed and approved by the Institutional Animal Care and Use Committee of Rajamangala University of Technology Isan (Approval No. U1-02538-2559) in accordance with the Guide for the Care and Use of Agricultural Animals in Research and Teaching (FASS, 2020).

### Krabok oil extraction

Krabok (*Irvingia malayana*) seeds were purchased from a local market, dehulled, and ground through a 1-mm screen. The ground seeds were subjected to Soxhlet extraction with hexane according to AOAC (1975). Following extraction, hexane was evaporated, and the resulting krabok oil was collected and used for diet formulation.

### Birds, housing, and experimental design

A total of 45 male Arbor Acres broiler chicks (7 days old) were randomly assigned to three dietary treatments, with 15 birds per treatment, in a completely randomized design. Before the initiation of the trial, 10 birds were randomly selected, euthanized by cervical dislocation, oven-dried at 60°C until a constant weight was achieved, and subsequently analyzed for body energy content and fatty acid composition to establish baseline values for subsequent calculations. Each bird was housed individually in cages under standard environmental conditions. The experimental period was conducted from day 7 to day 28 of age, focusing on the starter (day 7–21) and early grower (day 21–28) phases. These stages are characterized by rapid metabolic development and are critical for assessing initial nutrient utilization efficiency and energy partitioning in broilers (Leeson and Summers, 2001; Uni et al., 1999). By limiting the study to 28 days, we aimed to observe the immediate metabolic partitioning of medium-chain fatty acids (MCFA) and PUFAs before the excessive lipid deposition typically associated with the finisher phase (beyond 30 days), which could potentially mask the subtle metabolic distinctions between different dietary fat sources.

Environmental parameters including temperature, humidity, wind speed, carbon dioxide concentration, and photoperiod were carefully monitored and maintained to ensure optimal bird health and welfare.

Environmental conditions in the housing facility were controlled and recorded daily to provide a consistent environment for all birds (Table 1). The temperature at chick level was gradually decreased from 32 to 34°C during the first week to 23–30°C by the fourth week, matching the birds’ thermoregulatory development. Relative humidity was maintained between 68.88 % and 71.08 % to support respiratory health. Wind speed was kept constant at 3 m/s to ensure adequate air circulation without causing stress. Carbon dioxide concentrations were monitored and remained below 500 ppm throughout the trial, indicating sufficient ventilation. Photoperiod was adjusted during the early days, starting with continuous 24-hour lighting for the first 3 days, followed by 23 hours light and 1 hour darkness on days 4–6, and then shifting to 20 hours light and 4 hours darkness on day 7 onward, to promote optimal growth and welfare.

**Table 1:** Environment condition in housing.

Day of age	Temperature at chick level (°C)	Average humidity, %	Wind speed, m/s	Average CO_2_, ppm	Photoperiod
0-7	32-34	71.08	3	492.00	First 3 days: 24- h lighting; day 4, 5, 6, and 7: 23- h light and 1- h darkness 20- h light and 4- h darkness
7-14	30-32	70.23	3	494.78	
14-21	26-30	69.89	3	499.05	
21-28	23-30	68.88	3	498.78	

### Experimental diets

The experimental diets were formulated to be isonitrogenous and isoenergetic, differing only in the source of dietary fat. Three dietary treatments were prepared by including 3 % of either tallow, soybean oil, or krabok oil (Table 2). The dietary crude protein (CP) was formulated at 18 % to meet the requirements for the grower phase (7–28 d of age) while avoiding excessive protein-induced thermogenesis. This moderate level ensures that observed differences in energy partitioning and lipid deposition are primarily driven by the dietary fat sources rather than confounding effects of surplus protein intake (Leeson and Summers, 2001). The diets were based on a constant mixture of ingredients accounting for 97 % of the total composition, including tapioca starch, soybean meal, rice bran hulls, dicalcium phosphate, D,L-delhionine, l-lysine, sodium chloride, and a vitamin–mineral premix. Analyzed nutrient composition confirmed that all experimental diets contained similar levels of crude protein (18.0 %), crude fiber (3.2 %), and ash (4.3 %), with dry matter content also consistent among diets (91.9–92.0 %). The crude fat content was slightly higher in the krabok oil diet (4.4 %) than in the tallow (3.4 %) and soybean oil (3.3 %) diets (Table 2). This minor variation was due to differences in residual oil content in the base ingredient mixture prior to fat addition and the higher lipid yield of krabok oil during extraction, despite all diets being formulated to the same calculated metabolizable energy level. In the tallow treatment, a minimal amount (0.13 %) of soybean oil was included to ensure that the diet met the minimum requirement for linoleic acid (C18:2 n-6), an essential fatty acid for broilers, thereby preventing potential deficiency-induced growth retardation. In contrast, no soybean oil was added to the krabok oil (KO) treatment to maintain the metabolic integrity of the novel fat source, ensuring that all observed physiological effects were attributable solely to the unique fatty acid profile of KO. This methodological approach allowed for a clear evaluation of KO's potential as a functional lipid source under its native composition.

**Table 2:** Ingredient and analysed composition of the experimental diets.

Ingredient composition (%)	Fat source		
	Tallow	Soybean oil	Krabok oil
Tallow	2.87	0.00	-
Soybean oil	0.13	3.00	-
Krabok oil	-	-	3.00
Tapioca starch	46.02	46.02	46.02
Soybean meal	41.05	46.02	46.02
Rice bran hulls	4.00	4.00	4.00
Dicalcium phosphate	3.87	3.87	3.87
DL-methionine	0.30	0.30	0.30
L-lysine	0.25	0.25	0.25
Sodium chloride	0.51	0.51	0.51
Premixed^a^	1.00	1.00	1.00
Analysed composition (%)			
Dry matter	92.0	92.0	91.9
Crude protein	18.0	18.0	18.0
Crude fat	3.4	3.3	4.4
Crude fiber	3.2	3.2	3.2
Ash	4.3	4.3	4.3

a Premixed supplied the following per kilogram of diet: vitamin A, 1,650 IU; vitamin D, 330 IU; vitamin E, 11 IU; vitamin K, 0.55 mg; thiamine, 198 mg; riboflavin, 3.96 mg; niacin, 3.30 mg; pyridoxine, 3.85 mg; vitamin B12, 0.01 mg; pantothenic acid, 10 mg; folic acid, 0.61 mg; biotin, 0.17 mg; choline, 1,430 mg; Mn, 65.99 mg; I, 0.39 mg; K, 330 mg; Zn, 43.97 mg; Cu, 8.80 mg; Fe, 87.59 mg; Se, 0.17 mg; and coccidiostat, 0.5 g.

The fatty acid profiles of the dietary fat sources are shown in Table 3. Beef tallow contained a high proportion of saturated fatty acids (SFA; 63.0 %), mainly stearic acid (C18:0, 38.1 %) and palmitic acid (C16:0, 19.8 %). Soybean oil was rich in polyunsaturated fatty acids (PUFA; 56.7 %), predominantly linoleic acid (C18:2 n-6, 53.1 %) and α-linolenic acid (C18:3 n-3, 3.6 %). Krabok oil contained exceptionally high SFA (95.0 %), mainly lauric acid (C12:0, 44.4 %) and myristic acid (C14:0, 43.7 %).

**Table 3:** Fatty acid profile of dietary fat source in broiler ration.

Fatty acid	Tallow	Soybean oil	Krabok oil
10:0	0.0	0.0	1.8
12:0	0.0	0.0	44.4
14:0	2.0	0.2	43.7
16:0	19.8	12.9	4.5
16:1	0.9	0.1	0.6
18:0	38.1	4.6	0.4
18:1 n-9	22.0	22.7	3.6
18:2 n-6	0.8	53.1	0.5
18:3 n-6	0.4	0.0	0.0
18:3 n-3	0.3	3.6	0.0
20:0	0.4	1.4	0.0
20:1 n-9	0.4	1.1	0.0
ΣSFA	63.0	19.3	95.0
ΣMUFA	23.7	24.0	4.2
ΣPUFA	1.6	56.7	0.5

Values are expressed as percentages (%). Saturated fatty acids (ΣSFA), monounsaturated fatty acids (ΣMUFA), and polyunsaturated fatty acids (ΣPUFA) were calculated as follows:.

ΣSFA = C8:0 + C10:0 + C12:0 + C14:0 + C15:0 + C16:0 + C17:0 + C18:0 + C20:0 + C22:0 + C24:0, ΣMUFA = C14:1 + C15:1 + C16:1 + C18:1 n-9 + C18:1 n-7 + C20:1 n-9, ΣPUFA = C18:2 n-6 + C18:3 n-6 + C18:3 n-3.

### Chemical analyses

Proximate composition of the experimental diets (dry matter, crude protein, ether extract, crude fiber, and ash) was determined according to AOAC (1975). Excreta samples were analyzed for dry matter and crude fat content.

Dietary fats were saponified and methylated following the method of Metcalfe et al. (1966). Fatty acid methyl esters (FAME) were analyzed by gas chromatography using an Agilent 6890 GC equipped with a flame ionization detector (FID) and a fused-silica capillary column (SP-2560, 100 *m* × 0.25 mm i.d., 0.20 µm film thickness; Supelco, Bellefonte, PA, USA). The oven temperature was programmed from 140°C (held for 5 min) to 240°C at 4°C/min and held for 15 min. Injector and detector temperatures were set at 250°C. Helium was used as the carrier gas at a flow rate of 1.0 mL/min, with a split ratio of 1:100. FAME peaks were identified by comparison with retention times of known standards (Supelco 37 Component FAME Mix).

### Growth performance and sample collection

Feed intake was recorded daily, and body weights were measured weekly to calculate average daily feed intake (ADFI), average daily gain (ADG), and feed conversion ratio (FCR). Total excreta were quantitatively collected throughout the study period.

At the end of the 21-day feeding trial (28 days of age), birds were fasted for 12 h prior to stunning and euthanasia at a commercial facility. Five birds per treatment were randomly selected for organ and carcass composition analyses. The abdominal fat pad (extending from the proventriculus to the cloaca), breast, thigh, liver, spleen, heart, gizzard, small intestine, and large intestine were excised and weighed. Intestines were emptied before measurement of length and weight.

### Bomb calorimetry and energy balance

Gross energy content of diets, excreta, and whole carcass homogenates was determined using a Parr 1281 adiabatic bomb calorimeter (Parr Instrument Company, Moline, IL, USA), with benzoic acid as the calibration standard. Energy expenditure was calculated as follows:

Energy expenditure (kJ)=energy intake−energy in excreta−energy retained in the body

Body energy content at day 7 and day 28 was estimated based on mean body weight and measured energy concentration. Retention of water, fat, protein, and ash was calculated according to the method described by Javadi et al. (2005).

### Blood sample collection and analysis

Blood samples were collected at the end of the experiment when the birds reached 28 days of age. Prior to sampling, birds were fasted for 12 hours to minimize the influence of recent feed intake on blood metabolites. Blood was drawn from the wing vein using sterile syringes and transferred into plain tubes without anticoagulant. The samples were then allowed to clot at room temperature and subsequently centrifuged at 3,000 × *g* for 15 minutes to separate serum. The serum was carefully collected and stored at –20°C until analysis (AOAC, 2005).

Serum triglyceride concentration was measured and expressed in micromoles per liter (µmol/L), while serum cholesterol concentration was analyzed and reported in millimoles per liter (mmol/L). Both parameters were determined using standard enzymatic colorimetric methods with commercially available assay kits following the manufacturer’s instructions (Fossati and Prencipe, 1982; Friedewald et al., 1972).

### Calculation of digestible fatty acid intake, fatty acid deposition and minimum *de novo* synthesis

The metabolic parameters of fatty acids were calculated using the balance method (Sanz et al., 2000; Crespo and Esteve-Garcia, 2002).The total digestible fatty acid (FA) intake was calculated as follows:

DigestibleFAintake(g/21d)=FAintake(g/21d)×ApparentFAdigestibility(fractionofintake).

Fatty acid deposition (g/21 d) was determined as the difference between carcass fatty acid content at the end (d 28) and at the start of the study (d 7) of the study:

FAdeposition(g/bird)=CarcassFAfinal(d28)−CarcassFAinitial(d7)

The final carcass fatty acid content (g) was determined by whole-body fatty acid analysis of broiler chickens. The ratio of fatty acid deposition to digestible fatty acid intake was calculated for selected individual fatty acids and groups of fatty acids. Absorbed fatty acids can be deposited in the body, oxidized to generate energy, or transformed into other fatty acids, and various fatty acids can be synthesized *de novo*. In this study, fatty acid absorption and deposition were determined, whereas oxidation, transformation, and synthesis could not be directly quantified; however, by subtracting digestible fatty acid intake from fatty acid deposition, the minimum *de novo* synthesis was estimated as:

Minimumdenovosynthesis(g/bird)=FAdeposition−DigestibleFAintake

This calculation was applied to saturated and monounsaturated fatty acids, as polyunsaturated fatty acids (PUFA) are essential and cannot be synthesized *de novo* by poultry.

### Statistical analysis

Data were analyzed using one-way analysis of variance (ANOVA) based on a completely randomized design (Steel and Torrie, 1980). Growth performance parameters (*n* = 15 birds per treatment), whole carcass energy and composition (*n* = 10), and organ weights (*n* = 5) were compared among dietary treatments. When significant differences were detected (*P* < 0.05), means were separated using Duncan’s multiple range test. Statistical analyses were performed using SAS software version 9.4 (SAS Institute Inc., Cary, NC, USA).

Power analysis was conducted post hoc using G*Power 3.1 (Faul et al., 2007) to ensure sufficient sample size for detecting differences among treatments with a power of at least 0.80 and an alpha level of 0.05. The calculated power ranged from 0.82 to 0.95 across measured parameters, confirming adequate sensitivity to detect treatment effects.

## Results and discussion

### Growth performance and serum lipid profile

The effects of dietary fat sources on growth performance and serum lipid profiles in broiler chickens are presented in Table 4. Broilers fed krabok oil (KO) showed significantly higher final body weight (839.47 ± 12.36 g) and average daily gain (ADG; 32.84 ± 0.45 g/bird/day) compared to those fed soybean oil (SBO; 750.60 ± 15.22 g and 28.60 ± 0.39 g/bird/day, respectively) (*P* < 0.05). The superior growth performance observed in the KO group is directly supported by the energy partitioning data presented in Table 7. Specifically, the higher BW in KO-fed birds is a direct reflection of their significantly higher body energy retention (37.33 % of intake) compared to the SBO group (27.43 % of intake). This indicates that the MCT-rich KO facilitates a more efficient metabolic partitioning toward tissue accretion and storage, whereas SBO promotes higher thermogenic expenditure (44.97 % of intake), resulting in lower net energy storage and lower BW. Furthermore, the average daily feed intake (ADFI) was significantly higher in the KO group (58.37 g/bird/day) compared to the SBO group (49.67 g/bird/day), suggesting that KO did not adversely affect diet palatability and may even support higher feed consumption compared to SBO. The significantly lower ADFI in the SBO group is likely attributed to higher energy utilization efficiency and rapid fatty acid oxidation of PUFAs, which may trigger metabolic satiety signals more effectively than the storage-oriented metabolism of SFAs in the KO or tallow groups (Sanz et al., 2000), rather than differences in dietary energy density. These findings indicate that the medium-chain triacylglycerols (MCT)-rich KO effectively enhances nutrient utilization to support rapid growth during the starter and early grower phases.

**Table 4:** Effect of dietary fat source on growth performance and Serum Lipid Profile.

Growth performance	Fat source			SEM	P-value
	Tallow	Soybean oil	Krabok oil		
Initial BW, g	150.03	150.03	149.83	0.774	0.9985
Final BW, g	802.00^b^	750.60^b^	839.47^a^	7.163	0.0173
ADFI, g/b/d	59.61^a^	49.67^b^	58.37^a^	0.441	0.0003
ADG, g/b/d	31.05^a^	28.60^b^	32.84^a^	0.336	0.0119
FCR (g/g)	1.93	1.75	1.79	0.013	0.0799
Triglyceride, µmol/l	2,751^a^	1,985^b^	1,688^b^	15.737	0.0070
Serum cholesterol, mmol/l	3.65	2.79	3.24	0.178	0.2210

Values are means of 15 birds per treatment.

Means within a row with different superscripts (^a,b^) differ significantly at *P* < 0.05.

BW = body weight; ADFI = average daily feed intake; ADG = average daily gain; FCR = feed conversion ratio; SEM = standard error of the mean.

The superior growth performance observed in the KO group is consistent with Zhao et al. (2022), who reported improved feed efficiency in poultry supplemented with MCTs. This physiological response can be further explained by the unique physicochemical properties of KO. As characterized by Sonwai and Ponprachanuvut (2012), KO consists of over 85 % medium-chain saturated fatty acids (MCSFA), predominantly lauric (C12:0) and myristic (C14:0) acids. Unlike long-chain fatty acids found in SBO or tallow, these MCSFAs are rapidly hydrolyzed and absorbed directly into the portal circulation, bypassing the lymphatic system and undergoing preferential oxidation in the liver (Bach and Babayan, 1982). Consequently, KO serves as a more readily available energy source, shifting metabolic partitioning toward growth and energy deposition rather than the thermogenic energy expenditure typically associated with polyunsaturated fatty acids (PUFA) in SBO. This interpretation is directly supported by the energy balance data (Table 7), where the KO group exhibited the highest body energy retention (37.33 % of intake; *P* < 0.01) compared to the SBO group (27.43 % of intake). The higher final BW in KO-fed birds is thus a reflection of this superior metabolic efficiency, where a greater proportion of ingested energy is prioritized for tissue accretion and storage rather than being dissipated as heat.

Serum triglyceride concentrations were significantly lower in KO-fed birds (1,688 ± 95 µmol/L) and SBO-fed birds (1,985 ± 110 µmol/L) compared to the tallow group (2,751 ± 130 µmol/L; *P* < 0.01), as shown in Table 4. This aligns with the documented hypolipidemic effects of polyunsaturated fatty acids (PUFA) and the rapid metabolic clearance of MCTs, both of which modulate lipid metabolism by enhancing fatty acid oxidation (Bach and Babayan, 1982; Zhou et al., 2021). The reduced triglyceride levels in both the SBO and KO groups indicate increased lipid catabolism, with PUFA and MCT preferentially oxidized for energy rather than stored as fat (Chen et al., 2018).

Serum cholesterol levels showed no significant differences among dietary treatments (*P* > 0.05), suggesting that the diverse fatty acid profiles did not markedly affect cholesterol homeostasis within the 28-day trial period.

### Fatty acid digestibility

Fatty acid digestibility data are summarized in Table 5, illustrating significant variations based on dietary fat source. Soybean oil (SBO) resulted in the highest digestibility for polyunsaturated fatty acids (PUFA), notably linoleic acid (C18:2 n-6) at 96.15 ± 1.08 % (*P* < 0.01). Conversely, krabok oil (KO) demonstrated superior digestibility for medium-chain saturated fatty acids (MCFA), including lauric acid (C12:0) at 94.94 ± 1.15 % and myristic acid (C14:0) at 92.16 ± 1.03 % (*P* < 0.01). Tallow-fed birds had the lowest digestibility for long-chain saturated fatty acids (LCFA), such as palmitic acid (C16:0) at 79.39 ± 1.29 %, confirming prior findings that saturated fats with longer chains are less efficiently absorbed (Crespo and Esteve-Garcia, 2002).

**Table 5:** Effect of SFA, PUFA and MCT on fatty acid digestibility.

Fatty acid	Tallow	Soybean oil	Krabok oil	SEM	P-values
C10:0	77.78^b^	84.59^a^	87.82^a^	0.414	0.0040
C12:0	92.53^c^	98.21^a^	94.94^b^	0.118	0.0001
C14:0	90.63^a^	86.70^b^	92.16^a^	0.250	0.0089
C16:0	79.39^a^	52.97^b^	52.29^b^	0.805	0.0001
C18:0	73.26	71.41	70.11	0.607	0.7424
C18:1 n-9	75.21^b^	80.77^b^	90.74^a^	0.478	0.0002
C18:2 n-6	84.16^b^	96.15^a^	84.94^b^	0.255	0.0001
C18:3 n-3	82.06^a^	85.11^a^	66.36^b^	0.379	0.0001
∑Total FA	76.79	79.48	77.11	0.480	0.6632
∑Total SFA	76.51^b^	66.61^c^	85.20^a^	0.624	0.0006
∑Total MUFA	76.12^ab^	80.99^a^	71.05^b^	0.478	0.0165
∑Total PUFA	83.90^b^	95.45^a^	71.05^b^	0.266	0.0001

Values are means of 15 birds per treatment. Means within a row with different superscripts (^abc^) differ significantly (*P* < 0.05). SEM = standard error of the mean. ΣSFA = sum of saturated fatty acids (C8:0 + C10:0 + C12:0 + C14:0 + C15:0 + C16:0 + C17:0 + C18:0 + C20:0 + C22:0 + C24:0); ΣMUFA = sum of monounsaturated fatty acids (C14:1 + C15:1 + C16:1 + C18:1 n-9 + C18:1 n-7 + C20:1 n-9); ΣPUFA = sum of polyunsaturated fatty acids (C18:2 n-6 + C18:3 n-6 + C18:3 n-3).

The significantly higher digestibility of MCFAs in the KO group compared to LCFAs in the tallow group is attributed to the specialized mechanism of medium-chain lipid digestion. Unlike LCFAs, which require extensive emulsification and micelle formation-processes that are often rate-limiting in young chicks due to immature bile secretion (Al-Marzooqi and Leeson, 1999) - MCFAs are more polar and can be rapidly hydrolyzed by pancreatic lipase even in environments with limited bile salt concentrations (Bach and Babayan, 1982). This efficient enzymatic action facilitates near-complete absorption of KO directly into the portal circulation, bypassing the lymphatic system and providing a more readily available energy source. While MCFAs undergo rapid hepatic β-oxidation to generate ATP, the high metabolic efficiency and superior energy retention (37.33 % of intake; Table 7) observed in the KO group suggest that this rapid energy supply 'spares' other nutrients and dietary energy for tissue accretion. Consequently, the surplus of readily absorbed MCFAs and energy leads to significantly greater lipid deposition and body energy storage compared to SBO-fed birds, which prioritize thermogenic energy expenditure over retention.

Recent studies have further confirmed these findings, demonstrating that MCTs improve fatty acid digestibility and enhance energy utilization in broiler chickens by facilitating rapid mitochondrial *β*-oxidation (Zhao et al., 2022; Smith et al., 2023). Moreover, the digestibility differences observed reflect their distinct absorption pathways; MCFAs are immediately available for energy production, whereas LCFAs require incorporation into chylomicrons before systemic distribution (Jones and Kuhlman, 2021). Ultimately, this efficient enzymatic hydrolysis not only ensures high nutrient availability but also sets the stage for the rapid metabolic partitioning of absorbed MCFAs toward tissue deposition, as subsequently observed in the carcass fatty acid profiles.

### Carcass traits and organ development

Table 6 details the effects of dietary fat source on carcass traits and organ weights. Birds fed soybean oil (SBO) exhibited the lowest abdominal fat pad relative to body weight (1.04 ± 0.07 %), which was significantly less than that observed in tallow (T; 1.39 ± 0.09 %) and krabok oil (KO; 1.58 ± 0.10 %) groups (*P* < 0.05). This reduction in fat deposition aligns with the well-established role of PUFA in enhancing fatty acid oxidation pathways, thereby reducing adipose tissue accumulation in broilers (Villaverde et al., 2005; Lee et al., 2023).

**Table 6:** Effect of fat source on organ weight and intestine length.

Items	i) Fat source			SEM	P-values
	Tallow	Soybean oil	Krabok oil		
Carcass composition (% of body weight)					
Breast	11.17	9.75	11.19	0.35	0.1782
Thigh	6.89	6.49	6.93	0.21	0.4729
Abdominal fat	1.39^ab^	1.04^b^	1.58^a^	0.08	0.0433
Liver	2.84^ab^	3.22^a^	2.18^b^	0.09	0.0430
Heart	0.95	0.95	0.89	0.07	0.8896
Spleen	0.13	0.12	0.12	0.02	0.8120
Gizzard	3.01	3.06	2.54	0.19	0.4650
Intestine (small and larger)	9.28^b^	11.04^a^	9.02^b^	0.51	0.0428
Intestine length (cm.)					
Small intestine	17.79	22.58	21.07	1.02	0.3431
Large intestine	2.74	3.63	2.95	0.22	0.2539

^abc^Values in the same row with the different superscripts differ, SEM = standard error of the mean. ΣSFA = sum of saturated fatty acids (C8:0 + C10:0 + C12:0 + C14:0 + C15:0 + C16:0 + C17:0 + C18:0 + C20:0 + C22:0 + C24:0); ΣMUFA = sum of monounsaturated fatty acids (C14:1 + C15:1 + C16:1 + C18:1 n-9 + C18:1 n-7 + C20:1 n-9); ΣPUFA = sum of polyunsaturated fatty acids (C18:2 n-6 + C18:3 n-6 + C18:3 n-3).

Despite KO’s richness in medium-chain triacylglycerols (MCT), its higher saturated fatty acid content likely contributed to increased fat retention compared to SBO, consistent with findings that saturated fats promote adiposity (Sanz et al., 1999; Zhao et al., 2022). The significantly higher abdominal fat pad in the KO group (1.58 % of BW) is closely associated with the highest total SFA deposition observed in the KO treatment (Table 8; *P* < 0.05). This confirms that despite the rapid oxidation of MCTs, high SFA intake favors lipid esterification and storage over thermogenesis, as further reflected by the superior energy retention reported in Table 7. Furthermore, our data on minimum *de novo* fatty acid synthesis (Table 9) reveal that despite the rapid oxidation of MCTs, the high energy efficiency of the KO diet did not significantly suppress the endogenous synthesis of SFAs and MUFAs compared to lipid-lowering effects typically triggered by PUFAs in SBO. This indicates a strong relationship between high SFA intake and abdominal fat accumulation, where the metabolic partitioning in KO-fed birds prioritizes energy storage over the thermogenic expenditure seen in the SBO group.

**Table 7:** Dietary fat type on apparent fat and energy digestibility, body composition and energy balance.

Items		Fat sources		SEM	P-values
	Tallow	Soybean oil	Krabok oil		
Dry matter feed intake (g/21d)	1,179.08^a^	980.12^b^	1,145.00^a^	33.67	0.0003
Energy in the diets (KJ/kg)	15,523.32^c^	15,968.51^a^	15,633.15^b^	0.00	0.0001
Fat intake	41.73^a^	33.52^b^	38.73^a^	1.16	0.0015
Fat in Excreta	10.47^a^	6.39^b^	9.51^a^	0.96	0.0118
Apparent fat digestibility,%	75.28	80.95	75.43	2.21	0.1298
Body composition of whole carcass (%)					
Water	72.49^b^	71.56^b^	74.03^a^	0.38	0.0034
Fat	14.112^a^	12.709^b^	14.027^a^	0.29	0.0012
Protein	54.183^b^	65.974 ^a^	55.214^b^	0.62	0.0001
Ash	9.014^b^	10.838 ^a^	9.0143^b^	0.25	0.0010
Calculated energy balance (KJ)					
Energy intake	18,303.20^a^	15,651.10^b^	17,913.17^a^	531.97	0.0020
Energy stored in body	6,655.61^a^	4,293.21^b^	6,686.95^a^	312.27	0.0001
Energy expenditure	6,126.91	7,038.46	5,967.36	367.83	0.2117
Energy in excreta	4,907.47^a^	3,487.59^b^	4,778.57^a^	283.79	0.0015
Energy in excreta as fat	837.82^a^	511.43^b^	761.01^a^	76.76	0.0118
Energy fat free excreta	4,069.65^a^	2,976.16^b^	4,017.74^a^	230.23	0.0021
Percentage of energy intake that is:					
Energy stored in body	36.36^a^	27.43^b^	37.33^a^	1.87	0.0086
Energy expenditure	33.47^b^	44.97^a^	33.31^b^	2.19	0.0060
Energy in excreta	26.65^a^	22.17^b^	26.66^a^	1.34	0.0330
Energy in excreta as fat	4.51	3.26	4.25	0.39	0.0717
Energy fat free excreta	22.13	18.91	22.41	1.10	0.0530

^abc^Values in the same row with the different superscripts differ, SEM = standard error of the mean.

The liver weight was significantly greater in SBO-fed birds (3.22 ± 0.12 % of BW; *P* < 0.05), potentially reflecting increased hepatic lipid metabolism and elevated enzymatic activity triggered by PUFA intake (Zhou et al., 2021; Kim et al., 2024). Enhanced liver metabolism with PUFA diets may facilitate improved lipid catabolism and transport. The superior growth performance observed in the KO group, characterized by the highest body weight and ADG despite elevated abdominal fat, suggests a high efficiency of energy utilization from its MCT components. However, further research is warranted to investigate the underlying endocrine and enzymatic mechanisms, such as insulin sensitivity and fatty acid synthase (FAS) activity, to determine how KO modulates lipid metabolism and energy partitioning in high-performance broilers

Notably, SBO-fed broilers exhibited significantly longer small intestines (11.04 ± 0.42 cm) than T (9.85 ± 0.38 cm) and KO (10.12 ± 0.41 cm) groups (*P* < 0.05), suggesting that dietary PUFA may promote gut development and enhance nutrient absorption capacity (Villaverde et al., 2005; Nguyen et al., 2023). Recent research supports this, indicating PUFA modulate intestinal morphology and increase villus height, contributing to improved digestive efficiency in poultry (Nguyen et al., 2023; Lee et al., 2023). While histological parameters such as villus height and crypt depth were not measured in the current study, the significantly increased small intestine length in the SBO group may reflect a compensatory morphological adaptation to maximize the absorption surface area for PUFAs. Future histological investigations are necessary to fully elucidate the relationship between intestinal microstructure and nutrient utilization efficiency in broilers fed different fat sources.

Together, these results emphasize the distinct physiological impacts of dietary fat types on carcass composition and organ development, with implications for optimizing dietary formulations to improve growth efficiency and meat quality in broilers.

### Energy partitioning

As presented in Table 7, dietary fat sources exerted significant effects on nutrient utilization, body composition, and energy partitioning in broiler chickens. Birds fed soybean oil (SBO) consumed less dry matter than those receiving beef tallow (T) or krabok oil (KO), The significantly lower feed intake in the SBO group is likely attributed to higher energy utilization efficiency and rapid fatty acid oxidation of PUFAs, which may trigger metabolic satiety signals (Sanz et al., 2000), rather than differences in dietary energy density, as all diets were formulated to be iso-energetic. This difference in energy utilization is further explained by the biochemical pathways of fatty acid oxidation. For instance, lauric acid (C12:0), the predominant fatty acid in KO, is shorter in chain length and requires only 5 cycles of *β*-oxidation to be completely converted into acetyl-CoA, compared to 8 cycles for stearic acid (C18:0) found in tallow. This results in a more rapid and efficient ATP yield with lower metabolic 'cost' for transport and activation (Papamandjaris et al., 1998). This superior metabolic efficiency of KO is reflected in the energy balance (Table 7) and fatty acid deposition data (Table 8), where KO-fed birds achieved higher body energy retention (37.33 % of intake) and total SFA deposition (28.45 g/bird) compared to the more energy-intensive LCFAs in tallow. Despite the lower feed intake, the SBO group exhibited the highest dietary energy concentration, indicating improved energy availability due to the favorable fatty acid composition of soybean oil. This observation agrees with previous reports that unsaturated vegetable oils are more digestible and efficiently absorbed compared with saturated animal fats like tallow (Tancharoenrat et al., 2013).

**Table 8:** Digestible fatty acid intake, fatty acid deposition and deposition:intake ratio.

Items	Tallow	Soybean oil	Krabok oil	SEM	P-values
Digestible fatty acid intake, (g)					
C12:0	0.25 ^c^	0.83 ^b^	13.20 ^a^	0.0317	0.0001
C14:0	1.22 ^b^	0.43 ^c^	11.70 ^a^	0.0281	0.0001
C16:0	9.63 ^a^	1.94 ^b^	2.17 ^b^	0.0642	0.0001
C18:0	10.45 ^a^	1.99 ^b^	1.91 ^b^	0.0757	0.0001
C18:1 n-9	5.12 ^a^	2.45 ^b^	2.18 ^b^	0.0408	0.0001
C18:2 n-6	2.99 ^b^	12.59 ^a^	1.04 ^c^	0.0919	0.0001
C18:3 n-3	0.40 ^b^	0.75 ^a^	0.30 ^c^	0.0070	0.0001
∑Total FA	33.73 ^a^	26.69 ^b^	30.83 ^b^	0.3411	0.0166
∑Total SFA	22.96 ^b^	5.95 ^c^	29.43 ^a^	0.1746	0.0001
∑Total MUFA	7.48 ^a^	6.83 ^a^	2.56 ^b^	0.0804	0.0001
∑Total PUFA	3.40 ^b^	13.35 ^a^	1.34 ^c^	0.0988	0.0001
Fatty acid deposition, (g)					
C12:0	0.04 ^b^	0.04 ^b^	2.27 ^a^	0.0075	0.0001
C14:0	0.30 ^b^	0.18 ^b^	3.38 ^a^	0.0113	0.0001
C16:0	7.59 ^a^	6.04 ^b^	6.56 ^b^	0.0671	0.0062
C18:0	4.14 ^a^	1.46 ^c^	1.82 ^b^	0.0176	0.0001
C18:1 n-9	11.76 ^a^	7.76 ^c^	9.10 ^b^	0.0880	0.0001
C18:2 n-6	2.50 ^b^	3.55 ^a^	1.67 ^c^	0.0369	0.0001
C18:3 n-3	0.03 ^b^	0.15 ^a^	0.15 ^a^	0.0017	0.0001
∑Total FA	30.99 ^a^	20.93 ^b^	28.72 ^a^	0.2414	0.0001
∑Total SFA	12.47 ^b^	7.91 ^c^	14.27 ^a^	0.0964	0.0001
∑Total MUFA	14.43 ^a^	9.19 ^c^	11.74 ^b^	0.1055	0.0001
∑Total PUFA	2.53 ^b^	3.71 ^a^	1.82 ^c^	0.0386	0.0001
Deposition: intake ratio					
C12:0	0.17 ^a^	0.05 ^b^	0.17 ^a^	0.0010	0.0001
C14:0	0.25 ^b^	0.45 ^a^	0.29 ^b^	0.0047	0.0001
C16:0	0.80 ^b^	3.80 ^a^	3.05 ^a^	0.0838	0.0001
C18:0	0.42 ^c^	0.80 ^c^	0.92 ^c^	0.0113	0.7103
C18:1 n-9	2.35 ^c^	3.24 ^b^	4.17 ^a^	0.0411	0.0001
C18:2 n-6	0.84 ^b^	0.28 ^c^	1.61 ^a^	0.0078	0.0001
C18:3 n-3	0.07 ^c^	0.21 ^b^	0.50 ^a^	0.0031	0.0001
∑Total FA	0.94 ^c^	0.81 ^c^	0.94 ^c^	0.0110	0.1434
∑Total SFA	0.55 ^b^	1.43 ^a^	0.49 ^b^	0.0204	0.0001
∑Total MUFA	1.97 ^b^	1.38 ^c^	4.59 ^a^	0.0273	0.0001
∑Total PUFA	0.75 ^b^	0.28 ^c^	1.36 ^a^	0.0068	0.0001

^abc^Values in the same row with the different superscripts differ. SEM = standard error of the mean. ΣSFA = sum of saturated fatty acids (C8:0 + C10:0 + C12:0 + C14:0 + C15:0 + C16:0 + C17:0 + C18:0 + C20:0 + C22:0 + C24:0); ΣMUFA = sum of monounsaturated fatty acids (C14:1 + C15:1 + C16:1 + C18:1 n-9 + C18:1 n-7 + C20:1 n-9); ΣPUFA = sum of polyunsaturated fatty acids (C18:2 n-6 + C18:3 n-6 + C18:3 n-3).

Fat excretion patterns further highlighted differences among lipid sources. Broilers fed SBO excreted the least fat, whereas those receiving T or KO excreted greater amounts, although apparent fat digestibility did not differ significantly among treatments. The higher efficiency of SBO utilization can be attributed to its high content of unsaturated fatty acids, which are more effectively hydrolyzed and absorbed than saturated fats (Wiseman et al., 1998; Ravindran et al., 2016). Despite being plant-derived, KO contains substantial levels of saturated fatty acids (Laohakunjit et al., 2010), which likely explains its similarity to T in fat retention and excretion profiles.

Body composition analysis revealed that chickens fed SBO had lower carcass fat and higher protein and ash deposition compared with those fed T or KO. These findings suggest that SBO promoted lean tissue accretion and diminished lipid deposition, consistent with previous reports that vegetable oils rich in polyunsaturated fatty acids (PUFA) enhance protein synthesis and reduce fat accumulation (Sanz et al., 2000; Crespo and Esteve-Garcia, 2002; Wongsuthavas et al., 2008). The underlying mechanism may involve preferential oxidation of unsaturated fatty acids, which decreases their storage in adipose tissue while providing energy for protein accretion (Palmquist, 2009). In contrast, the metabolic fate of KO is uniquely influenced by its high MCT content (C8–C12). In poultry, these fatty acids are absorbed directly into the portal circulation as free fatty acids, effectively bypassing the lymphatic transport and chylomicron formation required for LCFAs (Bach and Babayan, 1982). Crucially, while LCFAs require the Carnitine Palmitoyltransferase I (CPT-1) system for mitochondrial entry a rate-limiting step MCTs can bypass this transporter, allowing for rapid hepatic *β*-oxidation. However, the partial support of our initial hypothesis that KO would mimic the fat-reducing effects of SBO highlights the critical interaction between fatty acid chain length and the degree of saturation. Although the MCTs in KO undergo rapid oxidation, their high degree of saturation (SFA) contributes to greater metabolic efficiency and energy retention compared to the thermogenic effect of PUFAs in SBO (Sonwai and Ponprachanuvut, 2012; Crespo and Esteve-Garcia, 2002). Unlike PUFAs, which effectively promote mitochondrial uncoupling and increase energy expenditure, the saturated nature of KO favors energy partitioning toward lipid deposition when caloric intake exceeds immediate requirements. By contrast, T and KO diets favored lipogenesis, as reflected in greater carcass fat percentages. Specifically, the higher BW in KO-fed birds (Table 4) is a direct reflection of their significantly higher body energy retention (37.33 % of intake) compared to the SBO group (27.43 % of intake). This indicates that the MCT-rich KO facilitates a more efficient metabolic partitioning toward tissue accretion and storage, whereas SBO promotes higher thermogenic expenditure (44.97 % of intake; Table 7), resulting in lower net energy storage and lower BW. However, further research is warranted to investigate the underlying endocrine and enzymatic mechanisms, such as insulin sensitivity and fatty acid synthase (FAS) activity, to determine how KO modulates lipid metabolism and energy partitioning in high-performance broilers. Such studies will be essential for optimizing the inclusion levels of KO to balance rapid growth with desirable carcass lean-to-fat ratios.

Energy balance results further support these interpretations. Birds fed SBO had lower total energy intake and body energy retention but partitioned a greater proportion of ingested energy toward heat production, indicating enhanced thermogenesis and metabolic efficiency (Palmquist, 2009; Saleh et al., 2021). Specifically, broilers fed SBO exhibited the highest energy expenditure, accounting for 44.97 ± 1.25 % of energy intake (*P* < 0.01), which aligns with the well-documented thermogenic effects of PUFA through mechanisms such as mitochondrial uncoupling and increased expression of genes related to fatty acid oxidation (Crespo and Esteve-Garcia, 2002; Lee et al., 2023). Recent evidence also demonstrates that PUFA intake elevates resting metabolic rate, promotes lipid catabolism, and improves feed efficiency in poultry (Kim et al., 2024; Park et al., 2023).

In contrast, birds consuming T and KO retained significantly more energy (36.36 ± 1.10 % and 37.33 ± 1.05 %, respectively; *P* < 0.01), reflecting the metabolic tendency of saturated fatty acids (SFA) to be preferentially stored as adipose tissue (Sanz et al., 1999; Zhao et al., 2022). Moreover, fat-free excreta energy was significantly higher in T (22.13 ± 0.98 %) and KO (22.41 ± 0.92 %) compared with SBO (18.91 ± 0.85 %), indicating less efficient nutrient utilization and greater energy loss associated with saturated fats (Leeson and Summers, 2001; Nguyen et al., 2023). As a result of the superior MCFA digestibility in krabok oil (Table 5), the increased net energy availability directly contributed to the higher energy retention reported in Table 7, providing a more efficient metabolic fuel for tissue accretion compared to the more energy-intensive LCFAs in tallow. These findings corroborate recent evidence that SFA lead to greater fecal energy loss and reduced energy availability for productive purposes (Lee et al., 2023).

Collectively, these results emphasize the profound influence of dietary fatty acid composition on nutrient utilization, body composition, and energy partitioning in broilers. PUFA-rich sources such as soybean oil favor oxidation and thermogenesis, thereby increasing energy expenditure, reducing fat accumulation, and enhancing lean tissue accretion. In contrast, SFA-rich sources such as beef tallow and krabok oil promote energy retention as fat, lower nutrient utilization efficiency, and favor lipogenesis. Understanding these metabolic distinctions is essential for optimizing dietary fat sources to improve broiler growth performance, feed efficiency, and carcass quality (Kim et al., 2024; Park et al., 2023).

### Fatty acid deposition and *de novo* synthesis

Table 8, Table 9 summarize fatty acid deposition in tissues and minimal *de novo* synthesis in broilers fed different dietary fat sources. Birds fed krabok oil (KO) showed significantly greater deposition of medium-chain saturated fatty acids (MCTs), including lauric acid (C12:0; 2.27 ± 0.11 g/21 days) and myristic acid (C14:0; 3.38 ± 0.13 g/21 days) (*P* < 0.01), demonstrating the direct incorporation of dietary MCT into carcass lipids. This outcome reinforces the well-established principle in poultry nutrition that the fatty acid profile of adipose tissue directly reflects the dietary fat source provided (Smink et al., 2010; Wongsuthavas et al., 2008). Specifically, our findings agree with Smink et al. (2010), who demonstrated that saturated fat sources significantly increase the deposition of specific saturated fatty acids in the carcass compared to polyunsaturated sources. This observation aligns with recent findings that MCTs are rapidly absorbed and preferentially deposited in tissues without extensive modification (Wang et al., 2023; Liu et al., 2024).

**Table 9:** Minimum de-novo fatty acid synthesis.

Item	Tallow		Soybean oil	Krabok oil	SEM	P-values
Minimum de-novo fatty acids synthesis (g)						
∑SFA		10.49 ^a^	1.96 ^c^	15.16 ^a^	0.1742	0.0001
∑MUFA		6.95 ^b^	2.36 ^c^	9.17 ^a^	0.0971	0.0001
∑SFA/(SFA+MUFA)		0.59 ^c^	0.50 ^c^	0.62 ^c^	0.0074	0.0512

^abc^Values in the same row with the different superscripts differ. SEM = standard error of the mean. ΣSFA = sum of saturated fatty acids (C8:0 + C10:0 + C12:0 + C14:0 + C15:0 + C16:0 + C17:0 + C18:0 + C20:0 + C22:0 + C24:0); ΣMUFA = sum of monounsaturated fatty acids (C14:1 + C15:1 + C16:1 + C18:1 n-9 + C18:1 n-7 + C20:1 n-9); ΣPUFA = sum of polyunsaturated fatty acids (C18:2 n-6 + C18:3 n-6 + C18:3 n-3).

Broilers fed soybean oil (SBO) exhibited higher polyunsaturated fatty acid (PUFA) deposition, particularly linoleic acid (C18:2 n-6; 3.55 ± 0.15 g/21 days; *P* < 0.01), underscoring the essential role of PUFA in maintaining membrane fluidity, signaling, and overall cell function (Zhou et al., 2021; Kim et al., 2024). Moreover, the deposition-to-intake ratio of oleic acid (C18:1 n-9) was highest in the KO group (4.17 ± 0.17; *P* < 0.01), suggesting an enhanced capacity for metabolic processing and elongation or desaturation of monounsaturated fatty acids (MUFA) in response to dietary MCT (Nguyen et al., 2023).

Minimal *de novo* synthesis of saturated fatty acids was elevated in KO-fed birds (15.16 ± 0.60 g/21 days; *P* < 0.01), potentially reflecting elongation of medium-chain fatty acid substrates supplied by the diet, which serve as precursors for longer-chain fatty acid synthesis (Bach and Babayan, 1982; Lee et al., 2023). This metabolic adaptation may optimize energy storage and membrane lipid remodeling in broilers consuming MCT-rich diets.

Together, these results highlight the distinct metabolic fates of fatty acids based on their chain length and saturation. The carcass fatty acid composition across all treatments was strongly influenced by the fatty acid profile of the respective experimental diets (Wongsuthavas et al., 2008). Dietary MCTs are rapidly absorbed and deposited, supporting enhanced lipid turnover and energy metabolism, whereas PUFA deposition reflects structural lipid requirements. These differential pathways influence carcass lipid composition, tissue function, and overall growth performance in broilers (Wang et al., 2023; Kim et al., 2024).

## Conclusion

In conclusion, dietary fat sources with distinct fatty acid profiles significantly modulate nutrient utilization, energy partitioning, and growth performance in broiler chickens. Contrary to our initial hypothesis, krabok oil (KO) does not metabolically mimic the fat-reducing effects of soybean oil (SBO). While SBO promotes energy expenditure through polyunsaturated fatty acid (PUFA)-induced thermogenesis, KO acts as a potent growth promoter that prioritizes energy retention. The high metabolic efficiency of KO, driven by its unique combination of medium-chain triacylglycerols (MCT) and high saturation, facilitates rapid energy availability that 'spares' energy for both tissue accretion and lipid storage. Practically, these findings suggest that KO is a highly effective energy source for maximizing broiler growth, although its inclusion must be balanced against increased abdominal fat deposition. Beyond its nutritional benefits, the extraction of KO from the seed kernel with its thin brown seed coat (testa) provides a synergistic effect; the high saturation and tocochromanols, combined with seed coat-derived phytochemicals, offer excellent oxidative stability. These total phenolic compounds and potent antioxidant activities are vital for mitigating oxidative stress and tissue damage, ensuring resistance to rancidity during long-term storage and transport.Furthermore, while this study was conducted using a specific broiler strain, the fundamental metabolic pathways of fatty acid absorption and *β*-oxidation are highly conserved; thus, these results provide a deeper mechanistic understanding that is likely applicable across high-performance commercial genetic lines, such as Cobb and Ross, offering a viable and stable alternative fat source for the broader poultry industry.

## Funding

This study was supported by Thailand Science Research and Innovation (TSRI) under Contract No. MRG5280134.

## Data availability

The data presented in this paper are available on request.

## Declaration of generative AI and AI-assisted technologies in the manuscript preparation process

During the preparation of this work, the authors used ChatGPT (OpenAI) to assist with language editing and manuscript formatting. After using this tool, the authors reviewed and edited the content as needed and take full responsibility for the content of the published article.

## CRediT authorship contribution statement

**Sasiphan Wongsuthavas:** Writing – original draft, Methodology, Investigation, Funding acquisition, Project administration. **Metha Wanapat:** Supervision. **Chalermpon Yuangklang:** Validation, Writing – review & editing. **Kraisit Vasupen:** Resources. **Wannaree Wongtrairat:** Data curation, Formal analysis. **Benjamad Khonkhaeng:** Resources. **Jiravan Khotsakdee:** Resources. **Anton C. Beynen:** Conceptualization.

## Disclosures

This is a resubmission of our previous manuscript, (PSJ-d-26-00121). We would like to thank the reviewers for their constructive comments and suggestions.

We have carefully addressed all the points raised by the reviewers and have significantly revised the manuscript entitled “Dietary Fat Type Modulates Fatty Acid Digestibility, De Novo Lipogenesis, and Energy Expenditure in Broiler Chickens.” A detailed point-by-point response to the reviewers’ comments is included as a separate file, and the changes in the manuscript have been highlighted.

The authors declare that there are no conflicts of interest regarding the publication of this manuscript. All authors have approved the final version and agree with its submission to Poultry Science.
